# Supplementary material for: Co-regulation of the transcription controlling ATF2 phosphoswitch by JNK and p38
Source: Nat Commun. 2020 Nov 13;11:5769. doi: 10.1038/s41467-020-19582-3 (PMC7666158; doi:10.1038/s41467-020-19582-3)
Supplement: Supplementary file 1 — Supplementary Information [file 41467_2020_19582_MOESM1_ESM.pdf]

Supplementary Information

Co-regulation of the transcription controlling ATF2 phosphoswitch by JNK and p38

Klára Kirsch[1], András Zeke[1], Orsolya Tőke[2], Péter Sok[1], Ashish Sethi[3], Anna Sebő[1], Ganesan Senthil Kumar[4] , Péter Egri[1], Ádám L. Póti[1], Paul Gooley[3], Wolfgang Peti[4], Isabel Bento[5], Anita Alexa[1], Attila Reményi[1,*]

[1]Biomolecular Interactions Research Group, Institute of Organic Chemistry, Research Center for Natural Sciences, H-1117 Budapest, Hungary.

[2]Laboratory for NMR Spectroscopy, Research Center for Natural Sciences, H-1117 Budapest, Hungary.

[3]Department of Biochemistry and Molecular Biology and Bio21 Molecular Science and Biotechnology Institute, University of Melbourne, Parkville, Victoria 3010, Australia

[4]Department of Chemistry and Biochemistry, University of Arizona, Tucson, USA.

[5]European Molecular Biology Laboratory, Hamburg, Germany

*Corresponding author: remenyi.attila@ttk.hu

Supplementary Table 1. Crystallographic data collection and refinement statistics

	JNK-ATF2(19-58)	pp-p38:ATF2(83-102)
**Data collection**		
Space group	P 1 21 1	P 2 21 21
Cell dimensions		
a, *b*, *c* (Å)	57.23 110.40 77.63	39.40 84.60 122.53
α, β, γ (°)	90.00 94.39 90.00	90.00 90.00 90.00
Resolution range (Å)	44.94 - 2.70	69.62 - 1.95
$CC_{1/2}$	99.6 (86.8)	99.9 (42.5)
R_{merge}†	6.2 (43.0)	7.8 (146.4)
$<I/\sigma(I)>$	10.3 (2.4)	17.8 (1.1)
Completeness (%)	91.4	98.9
Redundancy	3.3	12.5
No. reflections	23961	30380
**Refinement**		
$R_{work/}$ R_{free}	0.182 , 0.256	0.186, 0.220
No. atoms		
Protein	5971	2886
Ligand/ion	6	1
Solvent	125	351
B-factors (Å^2)		
Protein	77.5	43.32
Ligand	49.7	34.96
Solvent	66.1	52.94
Ramachandran		
Favored (%)	95	98
Allowed (%)	5	2
Outliers (%)	0	0
Rotamer outliers (%)	4	0
R.m.s deviations		
Bond lengths (Å)	0.012	0.008
Bond angles (º)	1.376	1.085
PDB ID	6ZR5	6ZQS

†$R_{merge} = \Sigma_{hkl}\Sigma_i |I_i(hkl)-<I(hkl)>|/\Sigma_{hkl}\Sigma_i I_i(hkl)$

Supplementary Table 2. Fitted parameters of the in silico model

unit	parameter	WT	WT +JNKi	S90N	S90N +JNKi	MUT4	fitted from	data
$\mu M^{-1}s^{-1}$	kon1	5.6	*	*	*	*	in vitro	FigS7a - pp-JNK
s^{-1}	koff1	19.6	*	*	*	*	in vitro	FigS7a pp-JNK
$\mu M^{-1}s^{-1}$	kon2	17.2	*	4.5x[a]	4.5x[a]	0.07x[b]	in vitro	FigS7a - pp-p38
s^{-1}	koff2	429	*	*	*	*	in vitro	FigS7a - pp-p38
$\mu M^{-1}s^{-1}$	kon3	0.429	*	*	*	*	assumption[c]	-
s^{-1}	koff3	429	*	*	*	*	assumption[c]	-
s^{-1}	k1	3.52	0	*	0	*	in vitro	FigS7a - pp-JNK
s^{-1}	k2	0.78	0	0	0	*	in vitro	FigS7b - p-S90
s^{-1}	k3	8.2	*	*	*	*	in vitro	FigS7a - pp-p38
s^{-1}	k4	1.67	*	*	*	*	in vitro	FigS7a - pp-p38
s^{-1}	keq7	4.70E-06	*	*	*	*	WB	Fig7b - pp-JNK
s^{-1}	kstim7	9.07E-05	*	*	*	*	WB	Fig7b - pp-JNK
s^{-1}	dp1	6.26E-04	*	*	*	*	WB	Fig7b - pp-JNK
s^{-1}	keq6	1.74E-05	*	*	*	*	NanoBit	Fig7b - p38-ATF2
s^{-1}	kstim6	1.16E-04	*	*	*	*	NanoBit	Fig7b - p38-ATF2
s^{-1}	dp2	1.76E-03	*	*	*	*	NanoBit	Fig7b - p38-ATF2
s^{-1}	dp3	9.54E-03	*	*	*	*	WB	Fig7b - pp-ATF2
s^{-1}	dp4	4.50E-03	*	*	*	*	NanoBit	Fig7b - p38-ATF2
µM	totJNK	1	*	*	*	*	assumption[d]	-
µM	totp38	2.65	*	*	*	*	estimated from WB[e]	Fig4a - pp-p38
µM	totATF2	1	*	*	*	*	assumption[d]	-

* same as in WT
[a] based on Ki values determined in vitro (Fig. 4b)
[b] based on enzyme kinetic data (Fig. 3c)
[c] based on enzyme kinetic data of the phosphorylation of ATF2(19-83) construct (0.5-100 uM) by p38. MM curve cannot be fitted in the applied concentration range, therefore binding affinity was assumed to be 1 mM. koff3 was set to the same as koff2.
[d] based on ref[1]
[e] To measure the NanoBit signal for p38-ATF2 TAD binding, cells were transfected with a heterologous p38 construct that increased the total in-cell concentration of the p38 enzyme. This was estimated based on quantitative WB data and heterologous p38 level was ~ 1.65 fold of the endogenous (which was assumed to be 1 µM).
in vitro: fitted to in vitro enzyme kinetics (quantitative dot-blot or phosphorimaging)
WB: fitted to cell-based Western-blots
NanoBit: fitted to in-cell luciferase complementation based protein-protein interaction data

Supplementary Table 3. List of primers.

Primer name	Primer sequence (5′-3′)
**PCR primers for E.coli constructs**	
ATF2-19-BamHI-Fw	CTGGGATCCATGAGTGATGACAAACCCTTTCTATGTACTGC
ATF2-100-NotI-Rev	TTTTTAAT GCGGCCGCT TGAAGCTTTCTTGAATTCATTCTCAAATGG
ATF2-78-NotI-Rev	AAACCCAC GCGGCCGCT GTTTTTCAAGAATCTTGTTGGTGTTGGGG
ATF2-58-NotI-Rev	ATTAGACT GCGGCCGCT TGCTGGACCAAATTTCAGTGTCATCTC
ATF2-50-NotI-Rev	CCAAATTT GCGGCCGCT CTCATGTTTATGTTTATGGACAGCCAAATGATCC
ATF2-Y-19-BamHI-Fw	TTATTAGGATCCTATAGTGATGACAAACCCTTTCTATGTACTGC
ATF2-58-XhoI-Rev	TATTTACTCGAGTCACTATGCTGGACCAAATTTCAG
cJUN-1-BamHI-Fw	TTATTAGGATCCATGACTGCAAAGATGGAAACGACCTTCTATGACG
cJUN-78-NotI-Rev	ATTAAATTGCGGCCGCTGCGCTCCAGCTCGGGC
ATF2-59-Not1-Fw	ATTAAATTGCGGCCGCAATGACAGTGTCATTGTGGC
ATF2-100-XhoI-Rev	TAATATCTCGAGTGAAGCTTTCTTGAATTCATTCTCAAATGG
ATF2-78-XhoI-Rev	TTATATCTCGAGTCAGTTTTTCAAGAATCTTGTTGGTGTTGGGG
ATF2-100-MUT4-NotI-Rev	TTATATTAGCGGCCGCTTGAAGCTTTCTTGGCTTCATTCTCAGCTGGACTCGC CAACTC
ATF2-106-XhoI-Rev	TATTAACTCGAGTCACTATTTTTTAATGTCATCTTCTGAAGCTTTCTTG
ATF2-19-CACA-BamHI-Fw	TTATTAGGATCCATGAGTGATGACAAACCCTTTCTAGCTACTGCGCCTGGAG CTGGCC
ATF2-90-NotI-Rev	TATTTATAGCGGCCGCTACTCGCCAACTCATTAAACAAACCC
ATF2-83-NotI-Rev	TATATATGCGGCCGCTACCCACTTCTTCACAGTTTTTCAAG
ATF2-83-XhoI_rev	TATTTACTCGAGACCCACTTCTTCACAGTTTTTCAAG
**Oligos for annnealed oligo cloning (with BamHI/NotI sites)**	
pepATF2(23-55)-Fw1	GATCCAAACCGTTTCTGTGCACCGCCCCGGGCTGCGGCCAGCGCTTTACCA ACGA
pepATF2(23-55)-Fw2	AGATCATCTGGCCGTGCATAAACATAAACATGAAATGACCCTGAAATTTGGC A-GC
pepATF2(23-55)-Rev1	GGCCGC-TGCCAAATTTCAGGGTCATTTCATGTTTATGTTT
pepATF2(23-55)-Rev2	ATGCACGGCCAGATGATCTTCGTTGGTAAAGCGCT
pepATF2(23-55)-Rev3	GGCCGCAGCCCGGGGCGGTGCACAGAAACGGTTT-G
pepATF2(23-50)-Fw1	GATCCAAACCGTTTCTGTGCACCGCCCCGGGCTGCGGCCAGCGCTTTACCA ACGA
pepATF2(23-50)-Fw1	AGATCATCTGGCCGTGCATAAACATAAACATGAAA-GC
pepATF2(23-50)-Rev1	GGCCGCAGCCCGGGGCGGTGCACAGAAACGGTTT-G
pepATF2(23-50)-Rev2	GGCCGCTTTCATGTTTATGTTTATGCACGGCCAGATGATCTTCGTTGGTAAAG CGCT
pepATF2(42-55)-Fw1	GATCC-CTGGCCGTGCATAAACATAAACATGAAA
pepATF2(42-55)-Fw2	TGACCCTGAAATTTGGCA-GC

pepATF2(42-55)-Rev1	ATGCACGGCCAG-G
pepATF2(42-55)-Rev2	GGCCGCTGCCAAATTTCAGGGTCATTTCATGTTTATGTTT
pepATF2(81-95)-Fw	GATCCGAAGTGGGTCTGTTTAACGAACTGGCCAGCCCGTTTGAAAACGAAAGC
pepATF2(81-95)-Rev	GGCCGCTTTCGTTTTCAAACGGGCTGGCCAGTTCGTTAAACAGACCCACTTCG
pep-cJun-Fw1	GATCCTACAGTAACCCCAAGATC
pep-c-Jun-Fw2	CTGAAACAGAGCATGACCCTGAACCTGGCTGACCCAAAGAAAAGCGGCCGC
pep-c-Jun-Rev1	CCCTGAACCTGGCTGACCCAAAGAAAAGCGGCC
pep-c-Jun-Rev2	TCATGCTCTGTTTCAGGATCTTGGGGTTACTGTAG
pep-c-JUN-K35E-Fw2	CTGGAACAGAGCATGACCCTGAACCTGGCTGACCCAAAGAAAAGCGGCCGC
pep-c-JUN-K35E-Rev2	TCATGCTCTGTTCCAGGATCTTGGGGTTACTGTAG
pepNFAT4-Fw	GATCCCTGGAACGCCCGAGCCGCGATCATCTGTATCTGCCACTGGAACCGAGC
pepNFAT4-Rev	GGCCGCTCGGTTCCAGTGGCAGATACAGATGATCGCGGCTCGGGCGTTCCAGG
**PCR primers for NanoBit assay**	
ATF2-19-XhoI-Fw	TTATCTCTCGAGCGGTATGAGTGATGACAAACCCTTTCTATGTAC
ATF2-100-NheI-Rev	TATTTAGCTAGCTGAAGCTTTCTTGAATTCATTCTCAAATGGAC
ATF2-MUT1-Rev	ATTAAGCTAGCTGAAGCTTTCTTGAATTCATTCTCAAATGGACTCGCCGCCTCATTAGCCAAACCCACTTC
ATF2-MUT2-Rev	TTATATGCTAGCTGAAGCTTTCTTGAATTCATTCTCAGCTGGACTCGCC
ATF2-MUT3-Rev	TATTTAGCTAGCTGAAGCTTTCTTGGCTTCATTCTCAAATGGACTCGC
ATF2-MUT4-Rev	TATAATGCTAGCTGAAGCTTTCTTGGCTTCATTCTCAGCTGGACTCGCCAACTC
ATF2-MUTΔ-Rev	TATTTAGCTAGCTGAAGCTTTCTTGAATTCATTCTCAAATGGACTCGCACAGTTTTTCAAGAATCTTGTTGGTGTTGGGG
ATF2-90-Rev	TATTTAGCTAGCACTCGCCAACTCATTAAACAAACCC
**PCR primers for GAL4 assay**	
GAL4-ATF2-19-BamHI-Fw	TATTTAGGATCCCAATGAGTGATGACAAACCCTTTCTATGTAC
GAL4-ATF2-100-Not1-Rev	TATTTATAGCGGCCGCTGAAGCTTTCTTGAATTCATTCTCAAATGG
GAL4-ATF2-19-CACA-BamHI-Fw	TATATAGGATCCCAATGAGTGATGACAAACCCTTTCTAGCTAC
**Primers for HT-MKK7-MLK3 and HT-MKK6EE**	
MKK7-HindIII-Fw	CTTAAGCTTGCCACCATGGCGGCGTCCTCCCTGGAAC
MKK7-BamHI-Rev	CCACCAGCACTGGATCCCCTGAAGAAGGGCAGGTGGGGC
linker-MLK3-BamHI-Fw	AGGGGATCCAGTGCTGGTGGTAGTGCTGGTGGTAGTGCTGGTGGTAGTGCTGGTGGTAGTGCTGGTGGTCTGCGGCTGGAGGAGGTGATC
MLK3-XhoI-Rev	TTGCTCGAGCAGCGCCTCCAACTGCTGCAGGAT
MKK6-HindIII-Fw	TATTTAAAGCTTGCCACCATGTCTCAGTCGAAAGGCAAGAAGCG

MKK6-NotI-Rev	TATTATTTAGCGGCCGCTGTCTCCAAGAATCAGTTTTACAAAAGATGC
**Primers for BiFC assay**	
ATF2-1-BamHI-Fw	TTATTAGGATCCATGAAATTCAAGTTACATGTGAATTCTGCCAGGC
ATF2-505-NotI-Rev	ATTAAATTGCGGCCGCTACTTCCTGAGGGCTGTGACTGG
cJ-UN-1-BamHI-Fw	TTATTAGGATCCATGACTGCAAAGATGGAAACGACCTTCTATGACG
c-JUN-331-NotI-Rev	ATTAAATTGCGGCCGCTAAATGTTTGCAACTGCTGCGTTAGCATGAG
**Mutagenesis primers**	
ATF2-K48E-Fwd	GGATCATTTGGCTGTCCATAAACATGAACATGAGATGACACTGAAATTTGGT CC
ATF2-K48E-Rev	GGACCAAATTTCAGTGTCATCTCATGTTCATGTTTATGGACAGCCAAATGATC C
cJUN-K35E-Fw	CTACAGTAACCCCAAGATCCTGGAACAGAGCATGACCCTGAACCTGGCC
cJUN-K35E-Rev	GGCCAGGTTCAGGGTCATGCTCTGTTCCAGGATCTTGGGGTTACTGTAG
ATF2-K48A-Fwd	GGATCATTTGGCTGTCCATAAACATGCACATGAGATGACACTGAAATTTGGT CC
ATF2-K48A-Rev	GGACCAAATTTCAGTGTCATCTCATGTGCATGTTTATGGACAGCCAAATGAT CC
ATF2-V44A-Fw	CGAGGATCATTTGGCTGCCCATAAACATAAACATGAGATGAC
ATF2-V44A-Rev	GTCATCTCATGTTTATGTTTATGGGCAGCCAAATGATCCTCG
ATF2-V44E-Fw	CGAGGATCATTTGGCTGAGCATAAACATAAACATGAGATGAC
ATF2-V44E-Rev	GTCATCTCATGTTTATGTTTATGCTCAGCCAAATGATCCTCG
ATF2-H47E-Fw	GGATCATTTGGCTGTCCATAAAGAGAAACATGAGATGACACTGAAATTTGG
ATF2-H47E-Rev	CCAAATTTCAGTGTCATCTCATGTTTCTCTTTATGGACAGCCAAATGATCC
ATF2-H47R-Fw	GGATCATTTGGCTGTCCATAAACGTAAACATGAGATGACACTGAAATTTGG
ATF2-H47R-Rev	CCAAATTTCAGTGTCATCTCATGTTTACGTTTATGGACAGCCAAATGATCC
ATF2-Q34R-Fw	GCCTGGATGTGGCCGGCGTTTTACCAACGAGG
ATF2-Q34-Rev	CCTCGTTGGTAAAACGCCGGCCACATCCAGGC
ATF2-CACA-Rev	GTAAAACGCTGGCCAGCTCCAGGCGCAGTAGCTAGAAAGGGTTTGTCATCA C
ATF2-L53E-Fw	CCATAAACATAAACATGAGATGACAGAGAAATTTGGTCCAGCACG
ATF2-L53E-Rev	CGTGCTGGACCAAATTTCTCTGTCATCTCATGTTTATGTTTATGG
ATF2-T69AT71A-Fw	GTCATTGTGGCTGATCAGGCCCCAGCACCAACAAGATTCTTGAAAAAC
ATF2-T69AT71A-Rev	GTTTTTCAAGAATCTTGTTGGTGCTGGGGCCTGATCAGCCACAATGAC
ATF2-S90A-Fw	GTTTGTTTAATGAGTTGGCGGCTCCATTTGAGAATGAATTCAAG
ATF2-S90A-Rev	CTTGAATTCATTCTCAAATGGAGCCGCCAACTCATTAAACAAAC
ATF2-S90N-Fw	GTTTGTTTAATGAGTTGGCGAATCCATTTGAGAATGAATTCAAG
ATF2-S90N-Rev	CTTGAATTCATTCTCAAATGGATTCGCCAACTCATTAAACAAAC
MKK6-EE-Fw	GTGGCTACTTGGTGGACGAGGTTGCTAAAGAAATTGATGCAGGTTGCAAAC C
MKK6-EE-Rev	GGTTTGCAACCTGCATCAATTTCTTTAGCAACCTCGTCCACCAAGTAGCCAC

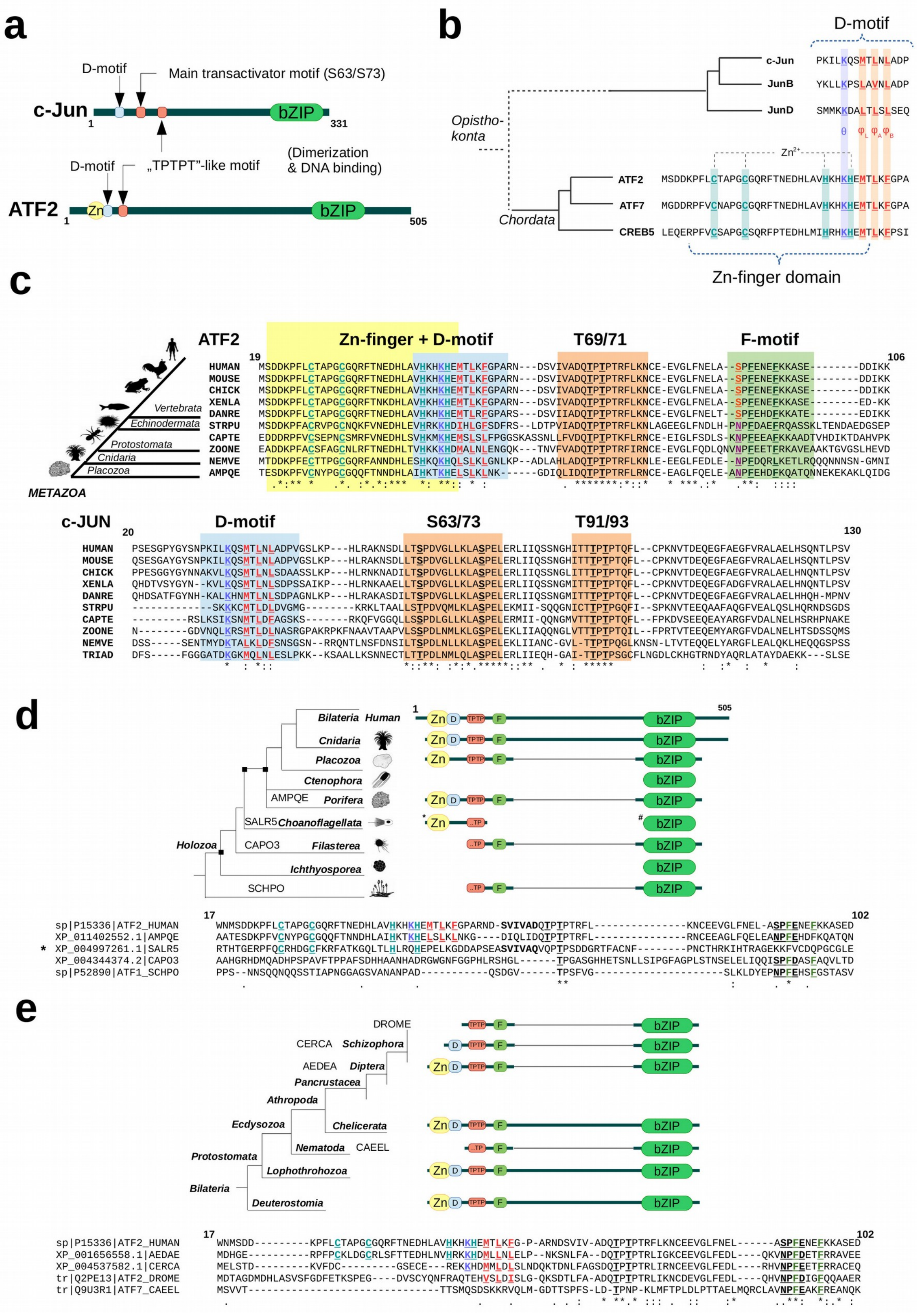

Supplementary Fig. 1. Evolutionary sequence conservation of AP-1 transcription factors (JUN and ATF).

a Schematic of c-Jun and ATF2. bZIP: basic region, leucine zipper domain – involved in dimerization and DNA binding; D-motif: docking motif – involved in MAPK binding; Zn: C2H2-

type Zn-finger domain – unknown function; TPTP-like motif: transactivator phosphoswitch – involved in phosphorylation-dependent upregulation of transcription. c-Jun contains different types of transactivator motifs: T91/T93 - similar to the TPTP-motif in ATF2 and S63/73 - unique in c-Jun. **b** Phylogenetic tree of different JUN and ATF proteins. In addition to a conserved D-motif, ATF proteins contain an N-terminal C2H2-type Zn-finger domain that overlaps with the basic region (θ) of the D-motif. φ – amino acids binding to different hydrophobic pockets of the MAPK docking groove; positively charged amino acids (θ) bind to the negatively charged common docking (CD) pocket. **c** Evolutionary sequence conservation of the transactivation region in ATF2 and c-Jun orthologs, respectively. The Zn-finger is colored yellow, the D-motif is blue, the transcription affecting MAPK target motifs, or phosphoswitches are orange and the F-motif is green. (TRIAD: *Trichoplax adhaerens* - Placozoa; AMPQE: *Amphimedon queenslandica* - sponge; NEMVE: *Nematostella vectensis* - sea anemone; ZOONE: *Zootermopsis nevadensis* - termite; CAPTE: *Capitella teleta* – worm; STRPU: *Strongylocentrotus purpuratus* - sea urchin; DANRE: *Danio rerio* – fish; XENLA: *Xenopus laevis* – frog; CHICK: Gallus gallus – bird; MOUSE: *Mus musculus*; HUMAN: *Homo sapiens*). **d** Co-occurrence of ATF2-type bZIP and ATF2 TAD elements in *Opisthokonta*. (Zn: C2H2 type zinc-finger, D: NFAT4 type docking motif, F: F-type S/NPFE/D..F motif). The analysis performed with BLASTp on Non-redundant protein sequence database using human sequences of ATF2, ATF7 and CREB5 as template. The thick line indicates a good match while the thin line indicates a poor match in the linker region between TAD and bZIP according to the BLASTp algorithm. Multiple alignments with selected sequences were performed using clustalX. (SALR5 – *Salpingoeca rosetta*, CAPO3 - *Capsaspora owczarzaki*, SCHPO - *Schizosaccharomyces pombe*). Note that *Salpingoeca rosetta* ATF2-TAD and bZIP module were found in two different transcripts. **e** Evolution of ATF2-type transcription factors in *Protostomata.* Loss of the Zn-finger+D-motif modul occured in *Nematoda* and gradually in *Diptera.* (CAEEL - *Caenorhabditis elegans*, AEDEA - *Aedes aegypti*, CERCA - *Ceratitis capitata*, DROME – *Drosophila melanogaster).*

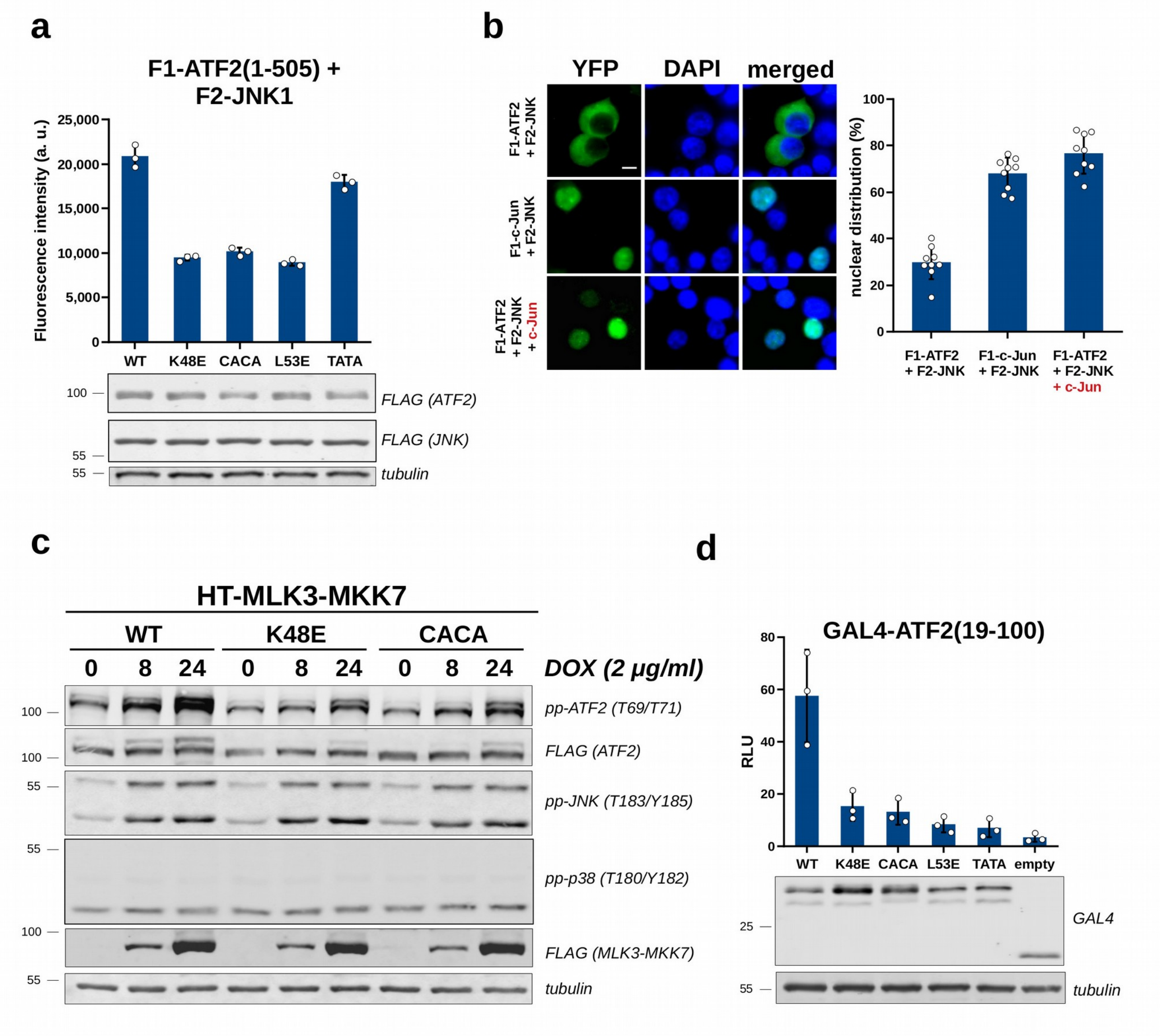

Supplementary Fig. 2. Characterization of the ATF2 Zn-finger+D-motif module in full-length proteins and in HEK293T cells.

a Bimolecular fluorescence complementation (BiFC) assay results with YFP fragment (F1 or F2) tagged full-length ATF2 constructs. JNK and ATF2 constructs were both FLAG-tagged and transiently transfected into HEK293T cells. The panel below shows the anti-FLAG Western-blot of cell lysates and shows comparable expression of the YFP fragment tagged constructs. Anti-tubulin antibody was used as load control. L53E: one of the key hydrophobic amino acids in the D-motif was mutated; TATA: T69 and T71 phosphorylation target sites were replaced to alanines. Error bars indicate SD calculated from three technical replicates. Data points are represented as mean values +/- SD. The experiment was repeated twice with similar results. **b** Cellular localization of ATF2-JNK1 (upper panels) or c-Jun-JNK1 (middle panels) complexes. Scale bar is 10 µM.The bottom panels show ATF2-JNK1 interaction when these BiFC probes were co-transfected with c-Jun (pcDNA-c-Jun). Nuclear localization of the BiFC signal is shown on the right. Nuclear distribution was calculated as the percentage of nuclear/total fluorescence. Error bars indicate SD of the nuclear distribution in cells (n=9) from each sample. Data points are represented as mean values +/- SD. **c** JNK-specific phosphorylation of full-length ATF2 constructs. HT-MLK3-MKK7 293T cells were

treated with doxycycline (DOX), then cells were lysed after the indicated time (0, 8 or 24 hrs), samples were loaded to SDS-PAGE gels and subjected to Western-blots (WB) using different antibodies. Western-blot results on lower panels show that addition of doxycycline promotes JNK phosphorylation (pp-JNK) but not that of p38 (pp-p38). Tubulin antibody is used as the load control, FLAG-MLK3-MKK7 WB signal shows the DOX-induced expression of the MLK3 kinase domain-MKK7 fusion construct that was FLAG-tagged. FLAG-ATF2 shows similar levels of the transfected ATF2 constructs, while pp-ATF2 WB signal shows the phosphorylation state of the ATF2 TAD phosphoswitch in full-length ATF2. The experiment was repeated independently at least three times with similar results. **d** Basal transcription activity of GAL4-ATF2 TAD constructs. The ATF2 TAD(19-100) was fused to the GAL4 DNA-binding domain (DBD) and transactivation was quantitatively assessed by using a GAL4 promoter/luciferase reporter plasmid. empty: GAL4 DBD transgene without TAD fusion. Error bars show SD calculated based on three independent experiments (n=3). Data points are represented as mean values +/- SD. The panel below shows similar levels of GAL4 fusion proteins and tubulin was used as sample load control. Source data are provided as a Source Data file.

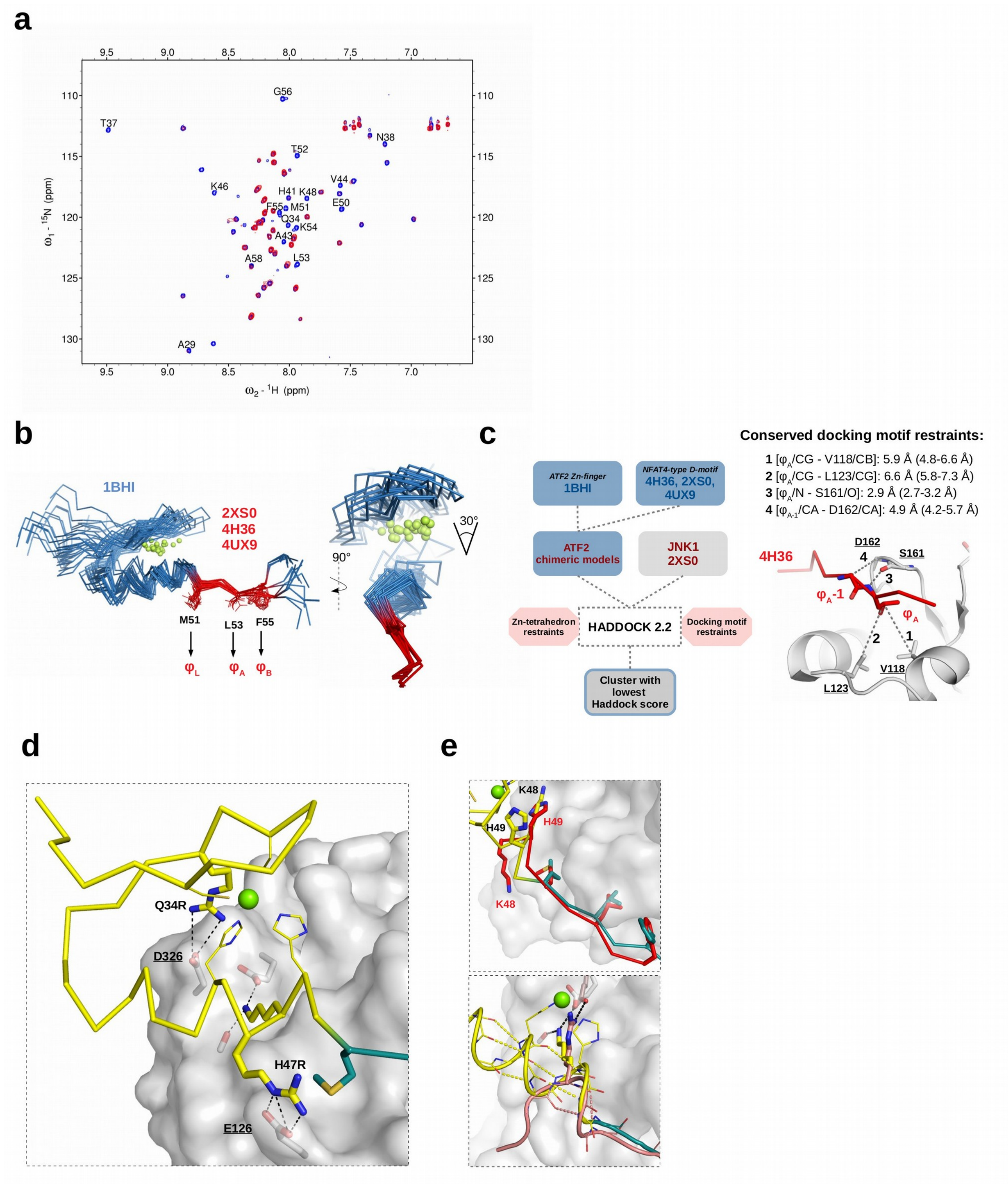

Supplementary Fig. 3. Details of the JNK-ATF2 NMR analysis and HADDOCK modeling.
a HSQC spectra of the ^{15}N-labeled ATF2(19-58) construct (blue: apo ATF2, red: JNK1-ATF2 1:1 molar ratio.) ^{1}H/^{15}N amide resonances of residues where I/Io < 0.1 are labeled. **b** Backbone trace of the ATF2(19-58) structural ensemble used for HADDOCK. In terms of the relative orientation of the Zn-finger and the D-motif, initial models deviated by ~30˚ around the axis set by the hydrophobic part of the D-motif. (The conformation of the hydrophobic region φ_L, φ_A, and φ_B were

built by using the conformation of peptides from known JNK-D-motif peptide crystallographic models (PDB ID: 2XS0, JNK1-pepNFAT4; 4H36, JNK3-pepATF2; 4UX9, JNK1: pepMKK7). (Zn-atom is shown as a green sphere.) **c** Schematic of HADDOCK input model construction. The NMR structural ensemble of the ATF2 Zn-finger (PDB ID: 1BHI) was manually connected to the D-motif region by using JNK:D-motif peptide complexes as a guide. The JNK1-pepNFAT4 crystal structure (PDB ID: 2XS0) shows the bound conformation of a D-motif with two amino acid placing between the θ and φ_L residues, which is the same spacing as in the ATF2 D-motif. This region is α-helical in the JNK1-pepNFAT4 structure and could be matched to the last turn of the α-helix from the ATF2 Zn-finger. During HADDOCK modeling two types of structural restraints were used: (1) Zn-tetrahedron C2H2 coordination restraints and (2) D-motif restraints for the hydrophobic region (4 structurally conserved atom-atom distances around φ_A). **d** Zoomed-in view of the Q34R and H47R region from the JNK1-ATF2(19-58) crystal structure. Both arginines allow the formation of new salt-bridges that increased binding affinity.**e** Structural comparison of the JNK1-ATF2(19-58) crystal structure with the JNK1-pepATF2[2] (top, in red) and JNK1-pepNFAT4[3] (bottom, in salmon) crystal structures. Top panel: Note the incorrect position of K48 and H49 if only a truncated D-motif peptide is used for crystallization[2]. Lower panel: Note the similarity between the NFAT4 peptide backbone and the alpha-helical region of the Zn-finger domain that holds and orients the positively charged residue towards the CD groove.

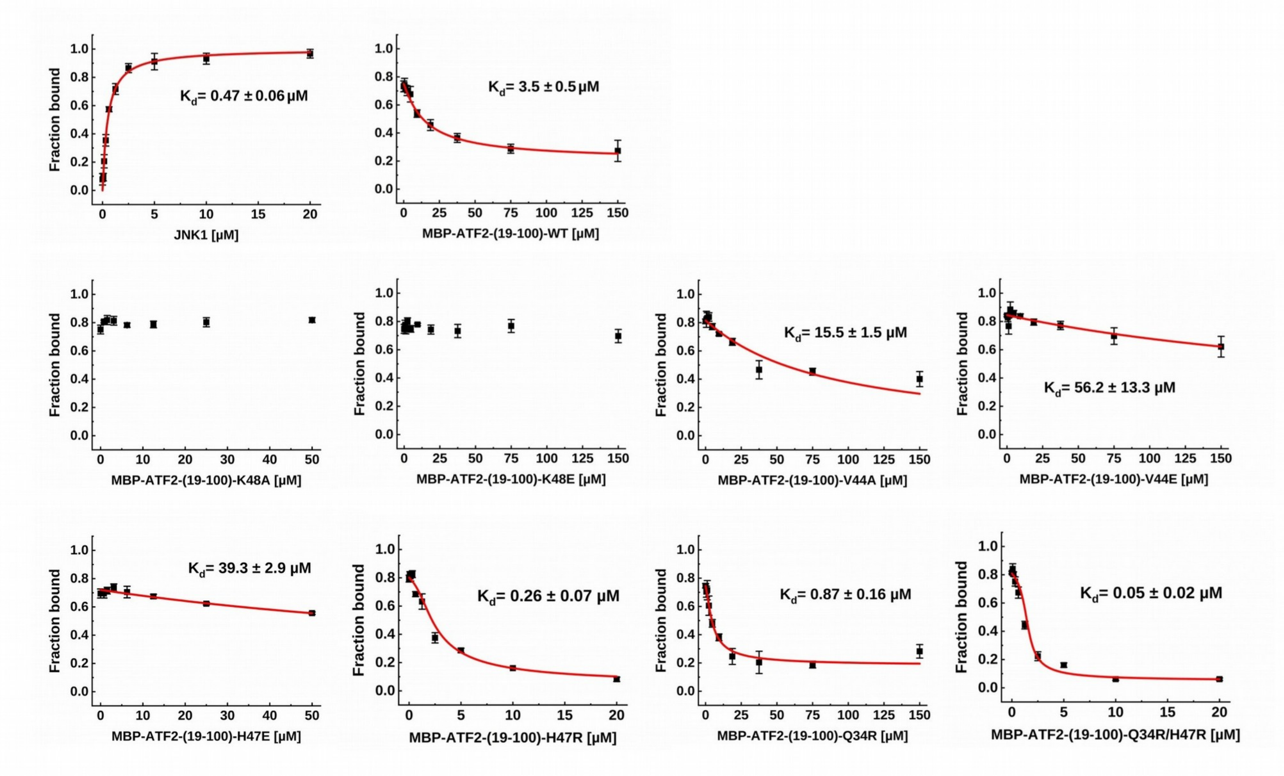

Supplementary Fig. 4. Results of fluorescence polarization (FP) based protein-protein binding assays.
The first panel shows the direct titration curve of JNK1 with a fluorescently labeled reporter docking peptide (CF-evJIP). The following panels show competitive titrations with purified MBP-ATF2(19-100)-WT and mutant constructs (WT: wild-type). Data were fit to a competition binding equation and errors indicate uncertainty in the fit. Error bars on the binding isotherms show SD based on three independent measurements (n=3). Data points are represented as mean values +/- S D. Source data are provided as a Source Data file.

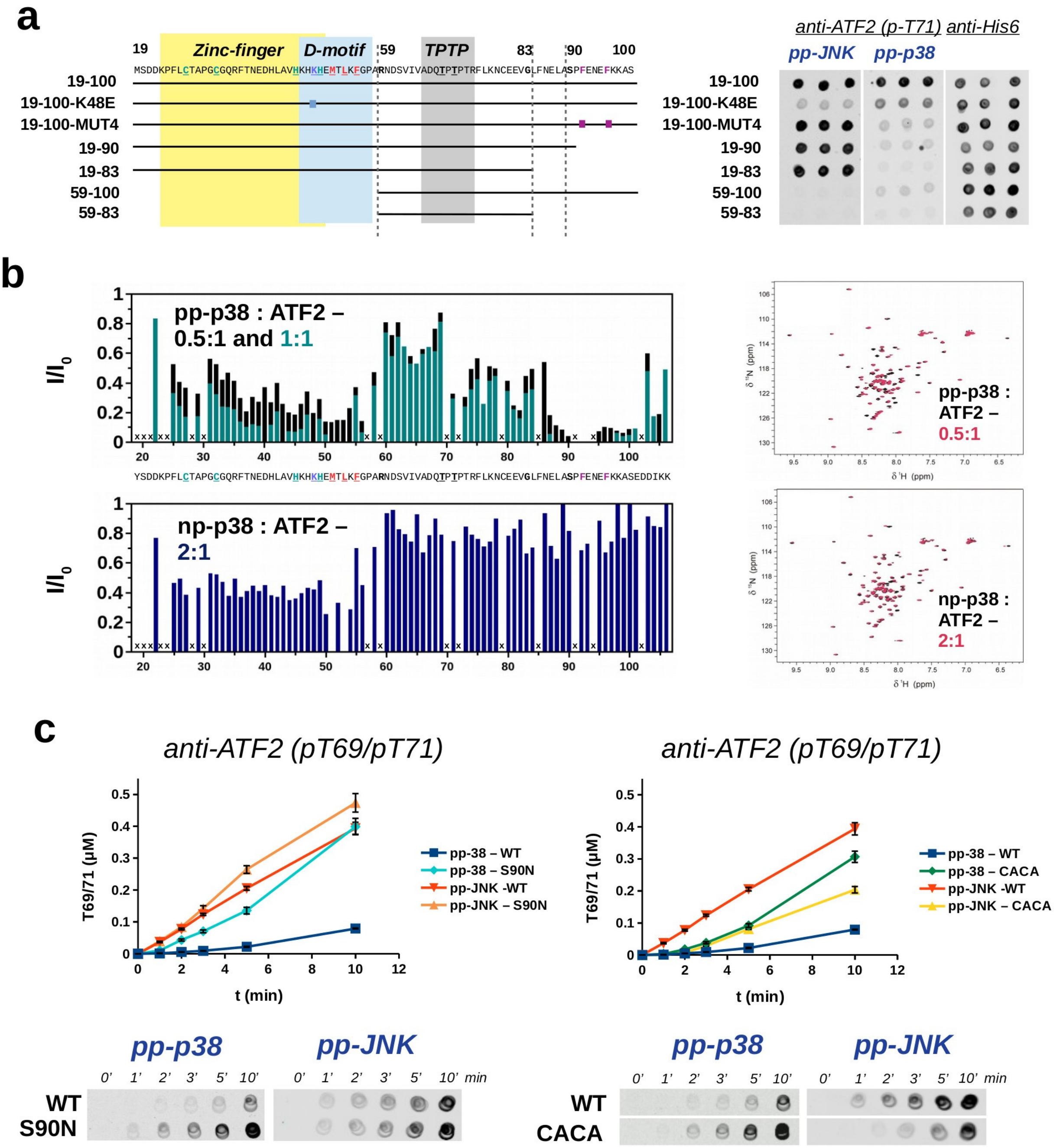

Supplementary Fig. 5. Characterization of p38-ATF2 binding.
a Phosphorylation of different ATF2 TAD constructs. Purified GST-ATF2 TAD-His6 fusion proteins were dotted on a membrane (three spots) and then incubated with pp-JNK1 or pp-p38. Equal load of the dots was checked by anti-His6 antibody, while target site phosphorylation was probed with anti-phosphoT71 antibody. **b** Interaction region mapping by NMR. 100 µM N^{15}-labelled ATF2(19-106) was mixed with pp-p38 in two different molar ratio (0.5:1 or 1:1) and intensity changes, normalized to the peak intensity without pp-p38, for ATF2 backbone amide HSQC peaks for the 0.5:1 (black) or 1:1 (cyan) pp-p38:ATF2 experiment were plotted. The lower panel shows the results similarly but when ATF2 was mixed with nonphosphorylated p38 in 2:1 molar ratio. ‘x’ indicates resonances of residues which are not resolved in the spectra therefore could not be quantified or proline The

HSQC spectra of the pp-p38:ATF2 (0.5:1) and np-p38:ATF2(2:1) are shown in the right. Black peaks indicate the apo spectra of ATF2, whereas titrated ATF2 peaks are colored with red.
c In vitro characterization of ATF2 TAD S90N and CACA mutants. Kinase assays were performed with 2 nM pp-JNK1 or 2 nM pp-p38 and 2 μM substrate. ATF2 TAD phosphorylation was detected by using anti-phosphoT69/71 antibody in dot-blots. Error bars indicate SD calculated from three te chnical replicates. Data points are represented as mean values +/- SD. (WT : wild type; CACA: two cysteines exchanged to alanines in the Zn-finger). Source data are provided as a Source Data file.

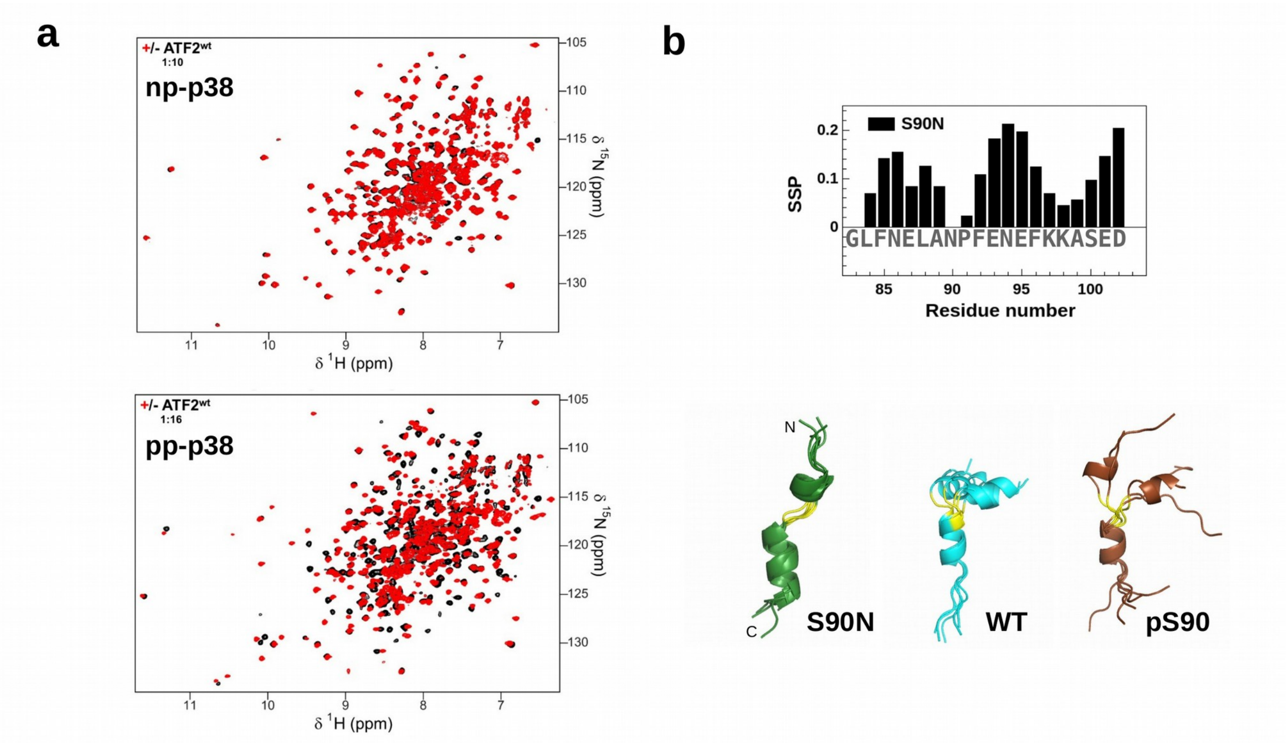

Supplementary Fig. 6. NMR analysis on SPFENEF motif peptides and their binding to p38. **a** 2D [^{1}H,^{15}N] TROSY NMR spectra of [^{2}H,^{15}N]-labeled nonphosphorylated p38 (np-p38) or double-phosphorylated p38 (pp-p38) alone (red) or in the presence of excess amounts (1:10 and 1:16 molar ratio, respectively) of the SPFENEF peptide (black). **b** Conformational analysis of SPFENEF peptides (S90N, WT and pS90) in solution. Secondary structure propensities (SSP) of peptides were calculated on the basis of H_α, C_α and C_β chemical shifts. The peptides are mostly disordered but have some α-helical propensity in solution and the lower panels display representatives of the 10 lo west-energy NMR structures calculated for the different ATF2(83-102) SPFENEF peptides. The re gion corresponding to the N/SP motif is colored yellow. Source data are provided as a Source Data file.

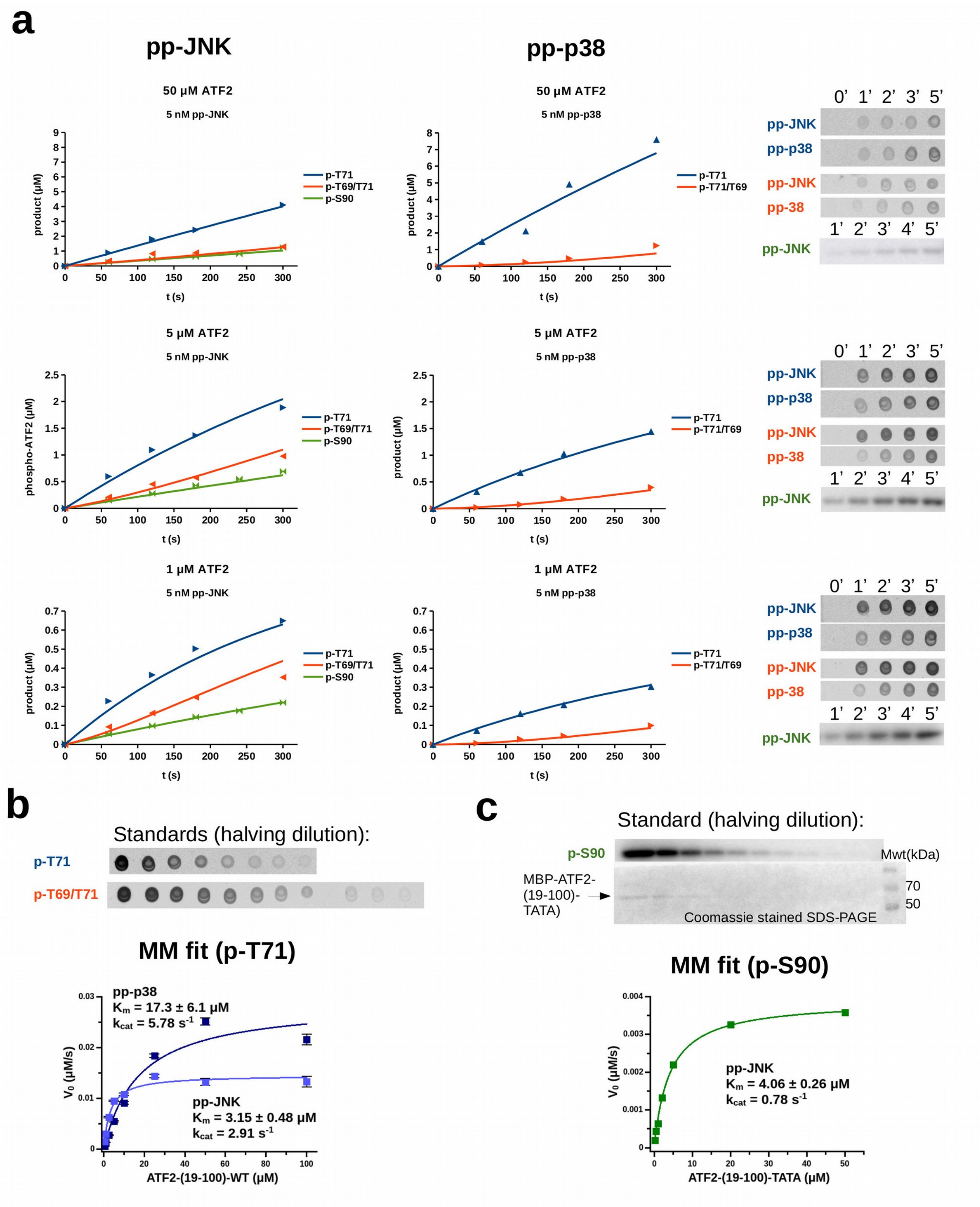

Supplementary Fig. 7. Parameter determination of JNK and p38 mediated ATF2 phosphorylation in vitro. a Reaction rates (k1, k3, k4) and binding rates ($k_{on}1$, $k_{off}1$, $k_{on}2$, $k_{off}2$) were fitted simultaneously to p-T71 and p-T69/T71 in vitro kinase assay results where various concentration (0.5-100 uM) of ATF2 substrates were used. Phosphorylation was detected by quantitative Western-blots (WB) using different phospho-specific antibodies, or by radioactive

phosphorimaging for pS90 (in green; see panel c). Graphs show different concentration examples of the kinetic data (points: dot-blot data, lines: BioNetGen model after fitting). Fitting gave reaction and binding rates for JNK (k1: 3.52/s, $k_{on}1$: 5.6/µM/s, $k_{off}1$: 19.6/s) and for p38 (k3: 8.02/s, k4: 1.67/s (for pT69→pT69/pT71), $k_{on}2$: 17.2/µM/s, $k_{off}2$: 429/s) with a derived binding affinity of ~ 3.5 µM and 25 ~ uM for JNK and p38, respectively. The rate of S90 phosphorylation by JNK (k2: 0.78/s) was obtained from the experiment shown in panel c; the kinetics of S90 phosphorylation can be modeled using $k_{on}1$ and $k_{off}1$ (green lines). Examples of the raw data are shown on the right.
b ATF2 phosphorylation at the phosphoswitch sites by pp-p38 and pp-JNK1. Upper panel: Determination of the linear range of the detection of p-T71 and p-T69/p-T71 for the dot-blot based kinase assay. 2X dilution series of fully phosphorylated ATF2 were used to confirm the linear range of detection. Lower panel: Michaelis-Menten (MM) fit of T71 phosphorylation by JNK and p38, based on WB dot-blot. Error bars show SD calculated based on three technical replicates. Data points are represented as mean values +/- SD. **c** ATF2 phosphorylation at S90 by pp-JNK1. Upper panel: Determination of the linear range for the phosphorimaging based quantification of S90 site p hosphorylation, and the same gel stained with Coomassie - with the molecular weight standards indi cated - is shown below. Lower panel: Michaelis-Menten (MM) fit of S90 phosphorylation by JNK, based on phosphorimaging data of the ATF2-TATA construct (TATA: T69 and T71 replaced top al anines). Source data are provided as a Source Data file.

Supplementary References

1. Kocieniewski, P., Faeder, J. R. & Lipniacki, T. The interplay of double phosphorylation and scaffolding in MAPK pathways. *J. Theor. Biol.* **295**, 116–124 (2012).
2. Laughlin, J. D. *et al.* Structural mechanisms of allostery and autoinhibition in JNK family kinases. *Structure* **20**, 2174–2184 (2012).
3. Garai, Á. *et al.* Specificity of linear motifs that bind to a common mitogen-activated protein kinase docking groove. *Sci. Signal.* **5**, ra74 (2012).
