# Supplementary material for: Co-regulation of the transcription controlling ATF2 phosphoswitch by JNK and p38
Source: Nat Commun. 2020 Nov 13;11:5769. doi: 10.1038/s41467-020-19582-3 (PMC7666158; doi:10.1038/s41467-020-19582-3)
Supplement: Supplementary file 3 — Description of Additional Supplementary Files [file 41467_2020_19582_MOESM3_ESM.docx]

Description of additional Supplementary Data files

File Name: Supplementary Software 1

Description: Computer-readable model implementation written in BNGL.
