# Supplementary material for: Co-regulation of the transcription controlling ATF2 phosphoswitch by JNK and p38
Source: Nat Commun. 2020 Nov 13;11:5769. doi: 10.1038/s41467-020-19582-3 (PMC7666158; doi:10.1038/s41467-020-19582-3)
Supplement: Supplementary file 5 — Reporting Summary [file 41467_2020_19582_MOESM5_ESM.pdf]

nature research

Corresponding author(s): Attila Reményi

Last updated by author(s): Oct 14, 2020

Reporting Summary

Nature Research wishes to improve the reproducibility of the work that we publish. This form provides structure for consistency and transparency in reporting. For further information on Nature Research policies, see our Editorial Policies and the Editorial Policy Checklist.

Statistics

For all statistical analyses, confirm that the following items are present in the figure legend, table legend, main text, or Methods section.

n/a	Confirmed	
☐	☒	The exact sample size (*n*) for each experimental group/condition, given as a discrete number and unit of measurement
☐	☒	A statement on whether measurements were taken from distinct samples or whether the same sample was measured repeatedly
☐	☒	The statistical test(s) used AND whether they are one- or two-sided<br>*Only common tests should be described solely by name; describe more complex techniques in the Methods section.*
☒	☐	A description of all covariates tested
☒	☐	A description of any assumptions or corrections, such as tests of normality and adjustment for multiple comparisons
☐	☒	A full description of the statistical parameters including central tendency (e.g. means) or other basic estimates (e.g. regression coefficient) AND variation (e.g. standard deviation) or associated estimates of uncertainty (e.g. confidence intervals)
☐	☒	For null hypothesis testing, the test statistic (e.g. *F*, *t*, *r*) with confidence intervals, effect sizes, degrees of freedom and *P* value noted<br>*Give P values as exact values whenever suitable.*
☒	☐	For Bayesian analysis, information on the choice of priors and Markov chain Monte Carlo settings
☒	☐	For hierarchical and complex designs, identification of the appropriate level for tests and full reporting of outcomes
☒	☐	Estimates of effect sizes (e.g. Cohen's *d*, Pearson's *r*), indicating how they were calculated

Our web collection on statistics for biologists contains articles on many of the points above.

Software and code

Policy information about availability of computer code

Data collection	P13 and P14 (Petra III) beamlines with XDS pipeline
Data analysis	Crystallography softwares: XDS (BUILT=20190315), CCP4i 7.1, COOT 0.9, PHENIX 1.18, Pymol 1.8<br>NMR softwares: NMRPipe 8.9, NMRFAM-SPARKY 1.412, Felix 2004, CCPNMR, Topspin 4.0.6., ARIA 2.2<br>Structure modeling: HADDOCK 2.2 webserver, MODELLER 9.24<br>Fluorescence microscopy image analysis: ImageJ 1.52a<br>Quantitative western blot analysis: Image Studio Lite 5.2<br>Rule-based signaling network modeling: BioNetGen 2.4, PyBioNetFit 1.1.1.<br>Data fitting and visualization: OriginPro 8, Qtiplot 0.9.8.9.<br>Statistical analysis and visualization: LibreOffice Calc 6.0.7.3

For manuscripts utilizing custom algorithms or software that are central to the research but not yet described in published literature, software must be made available to editors and reviewers. We strongly encourage code deposition in a community repository (e.g. GitHub). See the Nature Research guidelines for submitting code & software for further information.

Data

Policy information about availability of data

All manuscripts must include a data availability statement. This statement should provide the following information, where applicable:

- Accession codes, unique identifiers, or web links for publicly available datasets
- A list of figures that have associated raw data
- A description of any restrictions on data availability

The data that support this study are available from the corresponding author upon reasonable request. The crystal structure of the JNK-ATF2(19-58) and the pp-p38α:ATF2(83-102) complex were deposited in the Protein Data Bank (PDB) with the accession code 6ZR5 and 6ZQS, respectively. The following X-ray structures are available from the PDB: 1BHI, 2XS0, 2WO6, 6TCA. Source data are provided with this paper.

Field-specific reporting

Please select the one below that is the best fit for your research. If you are not sure, read the appropriate sections before making your selection.

☒ Life sciences ☐ Behavioural & social sciences ☐ Ecological, evolutionary & environmental sciences

For a reference copy of the document with all sections, see nature.com/documents/nr-reporting-summary-flat.pdf

Life sciences study design

All studies must disclose on these points even when the disclosure is negative.

Sample size	No sample size calculation was applied in this study to predetermine sample sizes for experiments using cell lines. A sample size of three was used as to evaluate the spread of the data and was determined based upon other studies with similar methodologies (PMID: 26538579, 19176525)
Data exclusions	No data were excluded from the analyses.
Replication	All experiments were replicated as stated in the figure legends
Randomization	Cells were randomly chosen for imaging and subsequent quantification on the experiment shown of Fig. S2b (single cell fluorescence microscopy). For other experiments samples were randomly chosen and cells were analyzed in bulk.
Blinding	No blinding, as the same investigator performed most experiments and analyzed the data

Reporting for specific materials, systems and methods

We require information from authors about some types of materials, experimental systems and methods used in many studies. Here, indicate whether each material, system or method listed is relevant to your study. If you are not sure if a list item applies to your research, read the appropriate section before selecting a response.

Materials & experimental systems

n/a	Involved in the study	
☐	☒	Antibodies
☐	☒	Eukaryotic cell lines
☒	☐	Palaeontology and archaeology
☒	☐	Animals and other organisms
☒	☐	Human research participants
☒	☐	Clinical data
☒	☐	Dual use research of concern

Methods

n/a	Involved in the study	
☒	☐	ChIP-seq
☒	☐	Flow cytometry
☒	☐	MRI-based neuroimaging

Antibodies

Antibodies used	anti-FLAG (Sigma, #F1804), anti-tubulin (Sigma, #T6199), anti-HIS (Sigma, #H1029), anti-phospho-T71 ATF2 (Cell Signaling, #9221), anti-phospho-ATF2-T69/T71 (Merck, #05-891), anti-GAL4 (Santa-Cruz, #sc-510), anti-phospho-JNK (Cell Signaling, #9251), anti-phospho-p38 (Cell Signaling, #9215), anti-phospho-c-Jun-S63 (Santa-Cruz, #sc-822), secondary anti-rabbit (IRDye® 800CW Goat anti-Rabbit IgG, Li-Cor, #926-32211), secondary anti-mouse (IRDye® 800CW Goat anti-Mouse IgG, Li-Cor, #926-32210), secondary anti-mouse (IRDye® 680RD Goat anti-Mouse IgG, Li-Cor, #926-68070)
Validation	All used antibodies were validated commercially. Certificates of analysis for the approved applications by the manufacturer and references are available on the company websites.<br>anti-FLAG (Sigma, #F1804): https://www.sigmaaldrich.com/catalog/product/sigma/f1804?lang=en&region=US

anti-tubulin (Sigma, #T6199): https://www.sigmaaldrich.com/catalog/product/sigma/t6199?lang=en&region=US
anti-HIS (Sigma, #H1029): https://www.sigmaaldrich.com/catalog/product/sigma/h1029?lang=en&region=US
anti-phospho-T71 ATF2 (Cell Signaling, #9221): https://www.cellsignal.com/products/primary-antibodies/phospho-atf-2-thr71-antibody/9221?Ntk=Products&Ntt=9221
anti-phospho-ATF2-T69/T71 (Merck, #05-891): https://www.emdmillipore.com/US/en/product/Anti-phospho-ATF2-Thr69-71-Antibody-clone-AW65,MM_NF-05-891
anti-GAL4 (Santa-Cruz, #sc-510): https://www.scbt.com/p/gal4-antibody-rk5c1
anti-phospho-JNK (Cell Signaling, #9251): https://www.cellsignal.com/products/primary-antibodies/phospho-sapk-jnk-thr183-tyr185-antibody/9251?Ntk=Products&Ntt=9251
anti-phospho-p38 (Cell Signaling, #9215): https://www.cellsignal.com/products/primary-antibodies/phospho-p38-mapk-thr180-tyr182-3d7-rabbit-mab/9215?Ntk=Products&Ntt=9215
anti-phospho-c-Jun-S63 (Santa-Cruz, #sc-822): https://www.scbt.com/p/p-c-jun-antibody-km-1
secondary anti-rabbit (IRDye® 800CW Goat anti-Rabbit IgG, Li-Cor, #926-32211): https://www.licor.com/bio/reagents/irdye-800cw-goat-anti-rabbit-igg-secondary-antibody
secondary anti-mouse (IRDye® 800CW Goat anti-Mouse IgG, Li-Cor, #926-32210): https://www.licor.com/bio/reagents/irdye-800cw-goat-anti-mouse-igg-secondary-antibody
secondary anti-mouse (IRDye® 680RD Goat anti-Mouse IgG, Li-Cor, #926-68070): https://www.licor.com/bio/reagents/irdye-680rd-goat-anti-mouse-igg-secondary-antibody

Eukaryotic cell lines

Policy information about cell lines

Cell line source(s)	HEK-293T (ATCC®, #CRL-3216™)
Authentication	The cell line was not authenticated
Mycoplasma contamination	The cell line was not tested for mycoplasma contamination.
Commonly misidentified lines (See ICLAC register)	no commonly misidentifies cell lines used in the study
